# Association of Primary Care Physicians Per Capita With COVID-19 Vaccination Rates Among US Counties

**DOI:** 10.1001/jamanetworkopen.2021.47920

**Published:** 2022-02-10

**Authors:** Chun-Han Lo, Leonard Chiu, Anna Qian, Muhammad Zarrar Khan, Hassan A. Alhassan, Axel J. Duval, Andrew T. Chan

**Affiliations:** 1 Clinical and Translational Epidemiology Unit, Massachusetts General Hospital and Harvard Medical School, Boston; 2 Division of Gastroenterology, Massachusetts General Hospital and Harvard Medical School, Boston; 3 Department of Epidemiology, Harvard T.H. Chan School of Public Health, Boston, Massachusetts; 4 Department of Medicine, Vanderbilt University Medical Center, Nashville, Tennessee; 5 Department of Internal Medicine, Yale New Haven Hospital, New Haven, Connecticut; 6 Department of Internal Medicine, Cleveland Clinic, Cleveland, Ohio; 7 Department of Medicine, University of Pittsburgh Medical Center, Pittsburgh, Pennsylvania; 8 Department of Medicine, Rutgers New Jersey Medical School, Newark; 9 Broad Institute of MIT and Harvard, Cambridge, Massachusetts; 10 Department of Immunology and Infectious Diseases, Harvard T.H. Chan School of Public Health, Boston, Massachusetts

## Abstract

This cross-sectional study examines the association between the number of primary care physicians (PCPs) per capita and COVID-19 vaccination rates among US counties.

## Introduction

COVID-19 vaccines have helped slow the spread of SARS-CoV-2 in the US. However, maximal population uptake of vaccines has been hindered by vaccine hesitancy. Greater participation of primary care physicians (PCPs) in vaccine distribution has been proposed as a strategy to combat vaccine hesitancy.^1,2^ Survey data suggest that a substantial portion of the unvaccinated population would be willing to get vaccinated if they had greater access to accurate information and receive encouragement from a trusted source.^3^ Primary care physicians can reach such individuals through direct engagement or alliances with community health workers, community centers, and mass-vaccination sites.^4^ Therefore, we sought to examine the association of the number of PCPs per capita with COVID-19 vaccination rates among US counties.

## Methods

We conducted a cross-sectional study of 2739 counties and county equivalents (herein termed *counties*) of the 3142 total counties in the US (87.2%) as of August 23, 2021. Eight California counties with populations less than 20 000 and 6 states (Alaska, Georgia, Hawaii, Vermont, Virginia, and West Virginia) were excluded because of insufficient vaccination data and missing variables. We linked the number of PCPs (including general family medicine, general practice, general internal medicine, and general pediatrics physicians) per 100 000 population for each county and other key variables with data on the percentage of the population who were fully vaccinated against SARS-CoV-2 obtained from the US Centers for Disease Control and Prevention and 3 state health departments (Colorado, Massachusetts, and Texas) (eTable in the Supplement). This study was deemed exempt by the Massachusetts General Hospital Institutional Review Board because it involved analysis of only publicly available aggregated data. This study followed the Strengthening the Reporting of Observational Studies in Epidemiology (STROBE) reporting guideline.

We examined the associations between the number of PCPs per 100 000 population and COVID-19 vaccination rates by using generalized estimating equation models with robust SEs after accounting for clustering within states and county population weights. The multivariable models were adjusted for demographic factors, urbanicity, socioeconomic status, and political leaning.^5^ We also conducted stratified analyses for metropolitan vs rural counties and Democratic vs Republican states (eMethods in the Supplement). All statistical analyses were performed using RStudio, version 1.4.1717. Two-sided *P* < .05 was considered statistically significant.

## Results

Among the 2739 US counties included in this study, PCPs were primarily concentrated in the Northeast, Florida, and many counties in the Midwest and West (Figure, A). This finding roughly corresponded to the distribution of counties with higher COVID-19 vaccination rates (Figure, B). After adjustment for potential confounders, counties in the highest decile of the number of PCPs per 100 000 population were associated with a 5.5% higher vaccination rate compared with those in the lowest decile (95% CI, 2.6%-8.4%) (Table). Every 10 additional PCPs per 100 000 population was associated with a 0.3% higher vaccination rate (95% CI, 0.2%-0.4%). In stratified analyses, we observed similar positive associations between the number of PCPs per 100 000 population and vaccination rates in rural areas or those with less than 2500 urban population (0.5% higher rate; 95% CI, 0.3%-0.7%) and in the 10 states with the highest Republican vote share (0.4% higher rate; 95% CI, 0.2%-0.6%).

**Figure.**
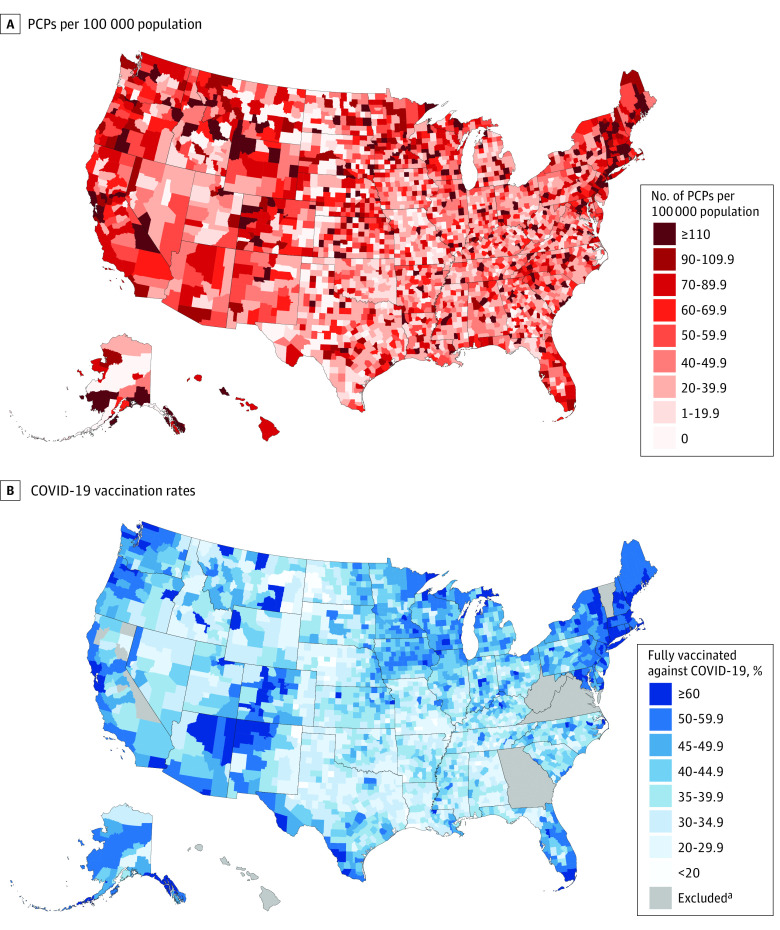
Number of Primary Care Physicians (PCPs) per 100 000 Population and COVID-19 Vaccination Rates Across US Counties A, Data on the number of PCPs per 100 000 population were available for all 3142 US counties. B, Data on COVID-19 vaccination rates were available for 2768 US counties (88.1%). ^a^Insufficient vaccination data.

**Table.** Differences in Vaccination Rates According to the Number of PCPs per 100 000 Population in the United States^a^

County stratification (No. of counties)	Difference in vaccination rate, % (95% CI)					
	By decile of PCPs per 100 000 population					Per 10 additional PCPs per 100 000 population
	Decile 1	Decile 5	Decile 7	Decile 9	Decile 10	
Overall (N = 2739)						
Median (IQR) No. of PCPs	0 (0-0)	39.7 (37.9-41.4)	55.4 (53.0-57.8)	85.2 (79.7-91.1)	121.5 108.7-148.4)	
Univariable model^b^	Reference	1.5 (−2.1 to 5.1)	2.9 (−0.7 to 6.5)	7.7 (3.9 to 11.5)	11.9 (7.7 to 16.2)	1.0 (0.8 to 1.3)
Multivariable model^c^	Reference	2.8 (0.3 to 5.3)	3.5 (0.9 to 6.1)	3.9 (0.8 to 7)	5.5 (2.6 to 8.4)	0.3 (0.2 to 0.4)
Large and medium metropolitan areas (n = 695)						
Median No. of PCPs	12.2 (6.3-15.2)	49.8 (47.9-52.5)	71.5 (69.5-74.9)	110.4 (101.0-116.1)	151.5 (142.4-175.0)	
Univariable model^b^	Reference	7.4 (3.0 to 11.8)	8.7 (5.6 to 11.8)	14.6 (11.0 to 18.2)	19.3 (16.4 to 22.3)	1.1 (0.8 to 1.3)
Multivariable model^c^	Reference	4.6 (1.7 to 7.6)	4.1 (0.6 to 7.5)	6.6 (3 to 10.1)	6.4 (2.9 to 9.9)	0.2 (0.1 to 0.4)
Rural areas or those with <2500 urban population (n = 566)						
Median No. of PCPs	0 (0-0)	19.3 (17.5-21.2)	35.8 (33.8-37.7)	65.1 (57.5-72.0)	110.6 (96.5-139.8)	
Univariable model^b^	Reference	0.9 (−1.7 to 3.4)	4.6 (2.4 to 6.9)	5.7 (2.0 to 9.3)	11.1 (6.6 to 15.6)	0.9 (0.6 to 1.3)
Multivariable model^c^	Reference	2.1 (−0.2 to 4.3)	3.5 (1.3 to 5.7)	5.8 (3.3 to 8.2)	6.6 (3.9 to 9.2)	0.5 (0.3 to 0.7)
10 States with the highest Democratic vote share (n = 328)^d^						
Median No. of PCPs	13.0 (0-19.4)	52.8 (51.0-54.4)	75.5 (72.1-78.5)	113.3 (102.9-120.3)	149.8 (135.5-168.8)	
Univariable model^b^	Reference	0.2 (−6.1 to 6.6)	5.2 (1.5 to 9.0)	9.1 (2.2 to 16.1)	14.2 (9.4 to 19.1)	1.2 (0.5 to 1.8)
Multivariable model^c^	Reference	−0.3 (−4 to 3.4)	1.4 (−1.3 to 4)	1.7 (−1.7 to 5)	1.9 (−1.4 to 5.3)	0.2 (−0.02 to 0.3)
10 States with the highest Republican vote share (n = 649)^e^						
Median No. of PCPs	0 (0-0)	35.9 (34.2-37.3)	48.1 (46.7-49.6)	69.5 (65.0-77.6)	111.9 (98.3-126.2)	
Univariable model^b^	Reference	2.4 (−0.9 to 5.8)	4.6 (1.9 to 7.2)	4.4 (1.6 to 7.1)	12.1 (8.7 to 15.5)	0.9 (0.6 to 1.2)
Multivariable model^c^	Reference	3.5 (0.7 to 6.2)	3.9 (0.9 to 6.8)	4.6 (1.6 to 7.5)	5.6 (1.6 to 9.6)	0.4 (0.2 to 0.6)

Abbreviation: PCPs, primary care physicians.

^a^ COVID-19 vaccination rates represent the percentages of population fully vaccinated against SARS-CoV-2 (those who have received the second dose in a 2-dose COVID-19 vaccine series or 1 dose of the single-shot Johnson and Johnson’s Janssen COVID-19 vaccine).

^b^ For univariable models, generalized estimating equation models with robust SEs were used after accounting for clustering within states and county population weights to estimate the associations between the number of PCPs per 100 000 population and COVID-19 vaccination rates.

^c^ Multivariable models were additionally adjusted for demographic factors (percentage of population aged ≥18 years, percentage of population aged ≥65 years, percentage male sex, percentage Hispanic residents, percentage non-Hispanic American Indian and Alaska Native residents, percentage non-Hispanic Asian residents, percentage non-Hispanic Black or African American residents, percentage non-Hispanic Native Hawaiian and Other Pacific Islander residents, percentage non-Hispanic White residents, and percentage of residents of non-Hispanic other race [ie, any race not already mentioned based on categories in US Census Bureau data]), urbanicity (population density, the Rural-Urban Continuum Codes), socioeconomic status (median household income, percentage population aged ≥25 years with a bachelor’s degree, unemployment rate, percentage of essential workers, uninsured rate), and political leaning (percentage Democratic vote share).

^d^ Based on greatest to least vote share, includes Massachusetts, Maryland, California, New York, Rhode Island, Connecticut, Delaware, Washington, Illinois, and New Jersey.

^e^ Based on greatest to least vote share, includes Wyoming, North Dakota, Oklahoma, Idaho, Arkansas, South Dakota, Kentucky, Alabama, Tennessee, and Utah.

## Discussion

In this cross-sectional study, we found that the number of PCPs per 100 000 population was independently associated with higher COVID-19 vaccination rates in the US. Our findings suggest that PCPs play a critical role in ensuring vaccine acceptance, especially in resource-limited and vaccine-hesitant regions, potentially through counseling and building local community trust and partnerships before they had access to vaccines.^6^ Limitations of the study include the accuracy of COVID-19 vaccine administration data, potential unmeasured confounders associated with the number of PCPs per capita and vaccination rates (eg, vaccine mandate), and the difficulty of disentangling our primary exposure from access to vaccines. Nevertheless, this study’s results provide support for expanding COVID-19 vaccine distribution to PCPs. Although fully incorporating PCPs into vaccination campaigns poses many challenges,^4^ PCPs may leverage their role as trusted messengers of scientific knowledge and educate communities about the importance of vaccination.

## Notes

Supplement. **eMethods.** Supplemental Methods **eTable.** Variable List and Data Sources
