# Supplementary material for: Association of Primary Care Physicians Per Capita With COVID-19 Vaccination Rates Among US Counties
Source: JAMA Netw Open. 2022 Feb 10;5(2):e2147920. doi: 10.1001/jamanetworkopen.2021.47920 (PMC8832176; doi:10.1001/jamanetworkopen.2021.47920)
Supplement: Supplement. — eMethods. Supplemental Methods eTable. Variable List and Data Sources [file jamanetwopen-e2147920-s001.pdf]

Supplemental Online Content

Lo CH, Chiu L, Qian A, et al. Association of primary care physicians per capita with COVID-19 vaccination rates among US counties. *JAMA Netw Open*. 2022;5(2):e2147920. doi:10.1001/jamanetworkopen.2021.47920

eMethods. Supplemental Methods

eTable. Variable List and Data Sources

This supplemental material has been provided by the authors to give readers additional information about their work.

© 2022 Lo CH et al. *JAMA Network Open*.

eMethods. Supplemental Methods

Covariate assessment

Covariates were grouped into four categories—demographic factors, urbanicity, socioeconomic status, and political leaning. Each category included one or more variables. The source of each variable can be found in **eTable**.

Demographic factors included the following variables: % population 18 years and above, % population 65 years and above, % male sex, % Hispanic, % non-Hispanic American Indian and Alaska Native, % non-Hispanic Asian, % non-Hispanic Black or African American, % non-Hispanic Native Hawaiian and Other Pacific Islander, % non-Hispanic White, and % non-Hispanic other race.

Urbanicity included population density and the Rural-Urban Continuum Codes. Population density was calculated by dividing the population by land area (people per square mile). The 2013 Rural-Urban Continuum Codes form a classification scheme that distinguishes metropolitan (metro) counties by the population size of their metro area and nonmetropolitan (nonmetro) counties by the degree of urbanization and adjacency to a metro area or areas. The codes range from 1 to 9, with 1 representing counties in metro areas of 1 million population or more, and 9 representing completely rural or less than 2,500 urban population, not adjacent to a metro area.

**2013 Rural-urban Continuum Codes**	
Code	Description
Metropolitan counties	
1	Counties in metro areas of 1 million population or more
2	Counties in metro areas of 250,000 to 1 million population
3	Counties in metro areas of fewer than 250,000 population
Nonmetropolitan counties	
4	Urban population of 20,000 or more, adjacent to a metro area
5	Urban population of 20,000 or more, not adjacent to a metro area
6	Urban population of 2,500 to 19,999, adjacent to a metro area
7	Urban population of 2,500 to 19,999, not adjacent to a metro area
8	Completely rural or less than 2,500 urban population, adjacent to a metro area
9	Completely rural or less than 2,500 urban population, not adjacent to a metro area

© 2022 Lo CH et al. *JAMA Network Open*.

Socioeconomic status included median household income, % population 25 years and older with a Bachelor's degree, unemployment rate, % essential workers, and uninsured rate.

Political leaning was defined by the percentage of eligible voters who voted for the Democratic Party in the 2020 US presidential election (termed Democratic vote share in this study).

All the variables mentioned above were included as continuous variables in model 2 in **Table** except for the Rural-Urban Continuum Codes (categorical).

Stratified analysis

We examined metropolitan vs rural counties. Metropolitan counties were defined as counties in metro areas of 250,000 or more population (Rural-Urban Continuum Codes 1 and 2), whereas rural counties were defined as completely rural or less than 2,500 urban population (Rural-Urban Continuum Codes 8 and 9).

We also examined 328 counties in ten states with the highest Democratic vote share and 649 counties in ten states with the highest Republican vote share. We did not include states for which we lacked vaccination data. In those cases, we used the next available state based on the ranking of vote share. Ten states with the highest Democratic vote share included Massachusetts, Maryland, California, New York, Rhode Island, Connecticut, Delaware, Washington, Illinois, and New Jersey. Ten states with the highest Republican vote share included Wyoming, North Dakota, Oklahoma, Idaho, Arkansas, South Dakota, Kentucky, Alabama, Tennessee, and Utah.

© 2022 Lo CH et al. *JAMA Network Open*.

eTable. Variable list and data sources		
**Variable**	**Data source**	**Source website**
**Main exposure**		
Number of primary care physicians per 100,000 population	Health Resources and Services Administration's Area Health Resources Files (2019-2020)	https://data.hrsa.gov/topics/health-workforce/ahrf
**Outcome**		
COVID-19 vaccination rate[a], %	Centers for Disease Control and Prevention COVID Data Tracker (2021)	https://covid.cdc.gov/covid-data-tracker/#datatracker-home
	Colorado Department of Public Health & Environment (2021)	https://covid19.colorado.gov/vaccine-data-dashboard
	Massachusetts Department of Public Health (2021)	https://www.mass.gov/info-details/massachusetts-covid-19-vaccination-data-and-updates#daily-covid-19-vaccine-report-
	Texas Department of State Health Services (2021)	https://dshs.texas.gov/coronavirus/immunize/vaccine.aspx
**Covariates**		
18 years and above, %	US Census Bureau American Community Survey (2015-2019)	https://data.census.gov/cedsci/
65 years and above, %		
Male, %		
Hispanic, %		
Non-Hispanic American Indian and Alaska Native, %		
Non-Hispanic Asian, %		
Non-Hispanic Black or African American, %		
Non-Hispanic Native Hawaiian and Other Pacific Islander, %		

© 2022 Lo CH et al. *JAMA Network Open*.

Non-Hispanic White, %		
Non-Hispanic other race, %		
Population, people	US Department of Agriculture (2019)	https://www.ers.usda.gov/data-products/county-level-data-sets/download-data/
Land area, square mile	US Census Bureau (2011)	https://www.census.gov/library/publications/2011/compendia/usa-counties-2011.html#LND
Rural-Urban Continuum Codes	US Department of Agriculture (2013)	https://www.ers.usda.gov/data-products/rural-urban-continuum-codes.aspx
Median household income, USD	US Census Bureau American Community Survey (2015-2019)	https://data.census.gov/cedsci/
Population 25 years and older with a Bachelor's degree, %		
Unemployed (in labor force), %		
Essential workers, %		
Uninsured rate, %		
Democratic vote share[b], %	MIT Election Data and Science Lab (2021)	https://electionlab.mit.edu/data

[a] COVID-19 vaccination rate represents the percentage of population fully vaccinated against SARS-CoV-2 (those who have received the second dose in a two-dose COVID-19 vaccine series or one dose of the single-shot Johnson and Johnson's Janssen COVID-19 vaccine).

[b] Based on the 2020 US presidential election.

© 2022 Lo CH et al. *JAMA Network Open*.
